# The Impacts of Dispositional Optimism and Psychological Resilience on the Subjective Well-Being of Burn Patients: A Structural Equation Modelling Analysis

**DOI:** 10.1371/journal.pone.0082939

**Published:** 2013-12-17

**Authors:** Fei He, Rong Cao, Ziqi Feng, Hao Guan, Jiaxi Peng

**Affiliations:** 1 School of Public Management, Northwest University, Xi'an, People's Republic of China; 2 School of Psychology, Beijing Normal University, Beijing, People's Republic of China; 3 Department of Burns and Cutaneous Surgery, Xijing Hospital, People's Republic of China; 4 Department of Psychology, Fourth Military Medical University, Xi'an, People's Republic of China; Rutgers University, United States of America

## Abstract

Burn wounds are severely stressful events that can have a significant impact on the mental health of patients. However, the impact of burns on individuals with different personality traits can be different. The present study aimed to investigate the impact of dispositional optimism on the subjective well-being of burn patients, and mainly focused on the confirmation of the mediator role of psychological resilience. 410 burn patients from five general hospitals in Xi'an accomplished the revised Life Orientation Test, Connor-Davidson Resilience Scale, and Subjective Well-Being (SWB) scale. The results revealed that both dispositional optimism and psychological resilience were significantly correlated with SWB. Structural equation modelling indicated that psychological resilience partially mediated the relationship between dispositional optimism and SWB. The current findings extended prior reports and shed some light on how dispositional optimism influenced SWB. Limitations of the study were considered and suggestions for future studies were also discussed.

## Introduction

With the gradual shift from biomedical models to biopsychosocial models, the interest in on patient well-being as a research area has increased, and modern medicine now is now focused on improving the quality of life while prolonging the lifespan of patients [1]–[4]. Burn wounds are a serious cause of stress and can significantly affect the mental health of patients. Statistics indicate that 10 to 44% of burn patients experience various forms of psychological symptoms or disorders during early trauma, 30 to 40% of patients continue to suffer significant long-lasting psychological disorders, and the incidence of post-traumatic stress disorder (PTSD) among burn patients ranges from 7 to 45% [5]–[7]. However, the effect of burns on individuals with different personality traits may vary. Using the Eysenck Personality Questionnaire, Holeva and Tarrier's survey of 256 burn patients revealed that individuals exhibiting neuroticism and low extraversion experienced high levels of anxiety; they found a strong correlation between individual anxiety levels and the occurrence of PTSD [8]. Although burn patients experience the same traumatic event, the psychological effects of the trauma differ according to individual personality traits. The current study presents a preliminary investigation into the effects of individual factors on the subjective well-being of burn patients, with a focus on two variables, namely, dispositional optimism and psychological resilience.

Originally proposed by Carver and Scheier, dispositional optimism is viewed as a stable psychological quality and a positive personality trait. It refers to an individual's positive expectations for the future [9]. Over the past 30 years, the body of research related to dispositional optimism has become increasingly rich and various groups have produced a large body of researches demonstrating correlations between dispositional optimism and subjective well-being, as well as its predictors. For instance, dispositional optimism has been found to positively correlate with self-esteem [10], [11], positive emotions [12]–[15], and life satisfaction [14], [16]–[17]. Negative correlations have also been reported between dispositional optimism and negative emotions [14]–[15], anxiety [18], and depression [16]–[17], [19]. These correlations have also been documented to be valid in many longitudinal studies [20]–[21] and cross-cultural studies [22].

Researches on psychological resilience began with the study on the responses to traumatic events. Research conducted by developmental psychopathologists on the formation and development of psychological resilience in the face of adversity has contributed to our understanding of why individuals do not succumb to stressful phenomena [23]–[24]. Block defines psychological resilience as “an individual's ability to change their behaviour to adapt to changing environmental trends and recover from stressful situations” [25]. As an individual's resource for coping with stress, psychological resilience can help effectively counter the negative effects of stress, and has therefore become a popular topic of research in positive psychology [26]. The positive influence of psychological resilience is reflected in many ways, for example, helping people recover from anticipated threat [27], improving an individual's ability to adapt to life and promoting individual development [28]. With the progression of research on psychological resilience, researchers have discovered psychological traits that are common among individuals with high psychological resilience, e.g. they are always very optimistic, feel that life is full of hope, and are curious about leading new lives [29]–[30]. With regard to the protective effect of psychological resilience on mental health, studies have mostly focused on the relationship between psychological resilience and negative mental health, such as depression, anxiety, loneliness, etc., and some researchers have also begun to focus on the relationship between psychological resilience and positive mental health, such as life satisfaction [28]. For instance, Chinese researchers Wu et al. reported a significant positive correlation between psychological resilience and life satisfaction among the families of earthquake victims [29].

In summary, there are enough reasons to believe that dispositional optimism and psychological resilience are positive predictors of subjective well-being, and that the two variables are positively correlated: It is usually the case that an optimistic person also has high psychological resilience. However, the trilateral relationship among the three variables remains unclear at present. Dispositional optimism is a stable psychological quality, psychological resilience is the ability to adapt to changing environments and recover from stressful situations, while subjective well-being is the overall affective and cognitive evaluation of quality of life. We hypothesize that people who have positive expectations for the future will have a stronger ability to withstand pressure and hence have a more positive evaluation of life. Using burn patients as research subjects, this study attempts to explore the impact of dispositional optimism and psychological resilience on post-traumatic subjective well-being.

## Method

### 2.1 Participants and Procedure

Participants were 410 burn patients (309 men and 101 women) from five general hospitals in Xi'an. All burns were due to scalds and second-degree burnt area covered 20–40%. Patients with head and face burns were excluded. The ages of burn patients ranged from 17 to 35, with a mean of 25.24 (SD = 2.76). Questionnaires were distributed at the second time when patients came to the hospital and participants completed the questionnaires in a separate room. Participants were told that they were engaging in a psychological investigation in which there were no correct or incorrect answers. Date collection lasted three months, from March to May, 2013. All participants provided informed consent before completing the measures (guardians on the behalf of the minors signed the informed consent) and received ¥50 in compensation. The research described in this paper meets the ethical guidelines of Xijing Hospital and has been approved by the ethics committee of the Fourth Military Medical University.

### 2.2 Instruments

#### 2.2.1 Dispositional optimism (Revised Life Orientation Test (LOT-R)

LOT-R, developed by Scheier, Carver and Bridges, is a 6-item measure (plus 4 filler items) of individual differences in dispositional optimism and pessimism. Items are rated from 1(strongly disagree) to 5(strongly agree) [32]. Examples of items include: “In uncertain times, I usually expect the best”, “If something can go wrong for me, it will”. Scale scores are the sum of items with reverse coding of relevant items. Higher scores reflect a greater tendency to expect more positive outcomes.

#### 2.2.2 Connor–Davidson Resilience Scale (CD-RISC)

The Connor-Davidson Resilience Scale (CD-RISC is a 25-item scale that measures the ability to cope with adversity [33]. Respondents rate items on a scale from 0 (not true at all) to 4 (true nearly all the time), and higher scores reflect greater resilience. Example items include: “I am able to adapt when changes occur”, “I can deal with whatever comes my way” and “I tend to bounce back after illness, injury, or other hardships.” A preliminary study of the psychometric properties of the CD-RISC in general population and patient samples supported its internal consistency, test–retest reliability, and convergent and divergent validity [33].

#### 2.2.3 Subjective well-being measures

Subjective well-being (SWB) is viewed as people's cognitive appraisal and emotional experience of life [34]–[35]. SWB scale was developed by Diener and Suh, including three sub-scales measuring life satisfaction, positive and negative effect [36]. The Satisfaction with Life Scale consists five items on a 7-point rating scale (from 1 =  strongly disagree to 7 =  strongly agree). Example items include: “In most ways my life is close to my ideal” and “I am satisfied with my life”. Scores are the sum of items with reverse coding of relevant items. Positive and negative effect scales were made up of 6 and 8 words respectively, each describe one kind of positive or negative emotion, like “angry”, “shameful”, “proud”, et al. Participants were asked to respond how often they were in these emotional state on 7-point rating scale (from 1 =  not at all to 7 =  all the time).

### 2.3 Data Analysis and the Test of Mediating Effect

To be sure of the structural relations of the latent structured model, a two-step procedure introduced by Anderson and Gerbing was adapted to analyses the mediation effect [37]. Firstly, the measurement model was tested to assess the extent to which each of the three latent variables was represented by its indicators. If the confirmatory measurement model is acceptable, then the maximum likelihood estimation would be used to test the structural model in AMOS 17.0 program. The following four indices were used to evaluate the goodness of fit of the model: (a) Chi square statistic (χ^2^), (b) the Standardized Root Mean Square Residual (SRMR), (c) the Root Mean Square Error of Approximation (RMSEA), and (d) the Comparative Fit Index (CFI) [38]–[39]. In this study, a model was considered to have a good fit if all the path coefficients were significant at the level of 0.05, SRMR was below 0.08, RMSEA was below 0.08, and CFI was 0.95 or more.

The mediating effect in the current study was tested for a significance by adopted the Bootstrap estimation procedure in AMOS. The reason for not using Sobel test, the commonly employed method for examining the statistical significance of a mediation effect, which involves computing the ratio of products of direct effects to their estimated standard error [40], is that Sobel test requires the products of direct effects follow a normal distribution which is always not accordance with the fact, thus resulted in the reduction of statistical efficacy [41]–[42]. The bootstrap test implemented by Preacher and Hayes tested the null hypothesis of insignificant indirect effect in another way. It takes the researcher's sample of size N and from it draws with replacement N values of independent, mediating and dependent variables to create a new sample. Repeat the option, for example, 1000 times, and then 1000 estimations of indirect effect estimations can be calculated [43]. The bootstrap test actually relies on the 95% confidence intervals from the empirical distribution of indirect effect estimates and Mackinnon suggested that the bootstrap method yields the most accurate confidence intervals for indirect effects [42], [44].

## Results

### 3.1 Measurement Model

Confirmatory factor analysis was used to exam whether the measurement model fit the sample data adequately or not. The measurement model included three latent constructs and 11 observed variables. An initial test of the measurement model came into being a satisfactory fit to the data: χ^2^ (39, N = 410) = 124.457, P<0.001; RMSEA = 0.07 and CFI = 0.957. All the factor loadings for the indicators on the latent variables were significant (P<0.001), indicating that the latent construct was well represented by its indicators.

Furthermore, as shown in table 1, correlations of the entire three latent variables, as dispositional optimism, psychological resilience and subjective well being (SWB) were significantly correlated with each other.

**Table 1:** Inter-correlations between dispositional optimism, psychological resilience and SWB.

	Mean	SD	1	2
1. Dispositional optimism	15.34	3.07		
2. Psychological resilience	67.77	11.66	0.237	
3. SWB	76.88	14.01	0.350	0.395

N = 410. All correlation coefficients are significant at p<0.01

### 3.2 Structural Model

In the first step, the direct effect of the predictor variable (dispositional optimism) on the dependent variable (SWB) without mediators was tested. The directly standardized path coefficient was significantly, β = 0.48, P<0.001. Then, a partially-mediated model (model 1) which contained mediators (psychological resilience) and a direct path from dispositional optimism to SWB was tested. The results showed that the model not very good fit to the data, χ^2^ (41, N = 410) = 136.929, P<0.001, RMSEA = 0.076, SRMR = 0.050 and CFI = 0.936. However, examination of parameter estimates revealed that the standardized path coefficient from dispositional optimism to SWB and psychological resilience, and from psychological resilience to SWB were all significant. Thus, according to the modification indices in the model 1, model 2 was created by add the correlations of residual terms between resilience1 and resilience2, resilience1 and resilience2.

**Figure 1:**
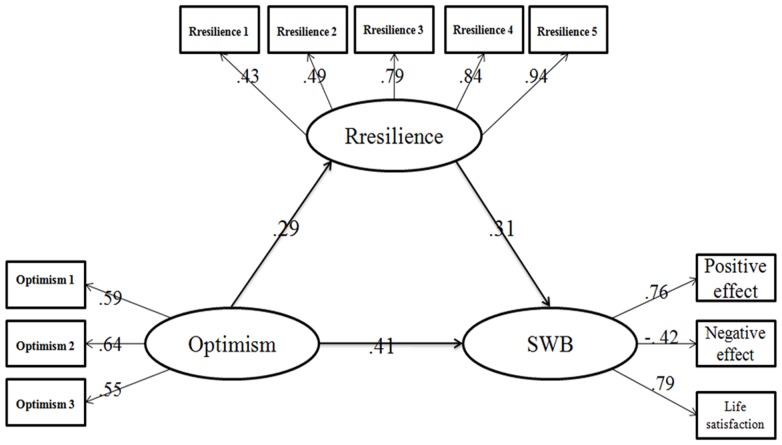
The full model of optimism, psychological resilience and subjective well-being.

After adding the correlations of the residual terms, the final meditational model, as shown in Fig. 1, was analyzed. The final meditational model showed a satisfied fitness to the data according to the following indices: χ^2^ (39, N = 410) = 90.246, P<0.001; RMSEA = 0.058; SRMR = 0.043; and CFI = 0.964. Taken together, those results showed the important role of psychological resilience in the relationship between dispositional optimism and SWB. The effect of dispositional optimism on SWB through psychological resilience was 17.9%.

Then the mediating effect was tested by adopted the Bootstrap estimation procedure (a bootstrap sample of 1,500 was specified).As shown in table 2, the direct and indirect effects and their associated 95% confidence intervals revealed that both dispositional optimism and psychological resilience had a direct effect on SWB, in addition, dispositional optimism had an indirect effect on SWB though psychological resilience.

**Table 2:** Direct and indirect effects and 95% confidence intervals for the final model.

Model pathways	Estimated effect	Lower bonds	Up bonds
**Direct effect**			
Optimism→Resilience	0.289	0.136	0.451
Optimism→SWB	0.411	0.268	0.552
Resilience→SWB	0.314	0.185	0.436
**Indirect effect**			
Optimism→Resilience→SWB	0.091	0.037	0.177

## Discussion

This study investigated the concurrent effect of dispositional optimism and psychological resilience on subjective well-being, and examined the mediator effect of psychological resilience on the relationship between dispositional optimism and subjective well-being of burn patients. This study found a positive relationship between optimism and SWB. This finding suggests that burn patients with high optimism are more likely to be capable of recovering from stressful situations and possess high subjective well being.

The finding that dispositional optimism and psychological resilience can positively influence subjective well-being is consistent with those of previous studies [14], [15], [17], [31]. Optimism is a positive psychological quality and individuals with higher dispositional optimism are more likely to have positive expectations of the future and view life events positively. Thus, they are highly satisfied with their life and are likely to amass more positive than negative experiences [13]–[16]. Psychological resilience is a capacity to recover from frustrations [25]. Individuals with high psychological resilience can easily adjust to the changing environment [26]. Both optimism and resilience are important components of psychological capital, which is regarded as a lasting and stable predictor of SWB [45]–[47]. Dispositional optimism and psychological resilience are therefore important protection factors of subjective well being.

Based on prior findings, this study mainly focused on the confirmation of the mediation effect of psychological resilience between dispositional optimism and subjective well being. Dispositional optimism and psychological resilience have always been found to be associated; in particular, optimistic individuals are generally known to be capable of recovering from frustrations [30]. Tugade, Fredrickson and Barrett suggested that personality traits are important factors of psychological resilience. Based on the results of the current work [48], we hypothesize that individuals with high dispositional optimism have a strong belief that good things will happen to them. They are thus convinced that a given situation is controllable, and that difficult times will be more convinced that the current situation is controllable and hard time will certainly pass. In other words, the positive expectation of the future is an important source of one's ability to overcome the current difficulties. Thus psychological resilience partially mediates the effect of dispositional optimism on subjective well being. According to the trait congruence effects, people with high positive effect related qualities tend to focus and process positive stimulus [49]. With this attention preference, individuals with high optimism comprehend and view life events in a positive way. Therefore, dispositional optimism can also influence SWB directly.

Burn patients were recruited as participants in the present work because their mental health is known to be influenced greatly by the stress resulting from their burn wounds. The results suggest that although burn patients experience the same traumatic event, those with higher dispositional optimism and stronger psychological resilience will feel little mental suffering and are more likely to recover. To improve the subjective well being and life quality of burn patients, we should adopt interventions that primarily focus on increasing the dispositional optimism and psychological resilience of these patients.

In sum, this study provides insights into the relationships among dispositional optimism, psychological resilience and subjective well being. Dispositional optimism acts as a protective factor by increasing the ability of an individual to recover from frustrations. Such ability has a beneficial effect on SWB. Nevertheless, this study has certain limitations. First, personality is always dependent on culture. As all measures used in this study originate from western countries, some confounding factor caused by cultural difference may be induced. Second, some recent studies have suggested that dispositional optimism is bidimensional and consists of optimism and pessimism factors [50], [51]. In the present study, the Cronbach's alpha coefficient for the LOT-R was only at the barely acceptable level. Considering that this study is only an initial exploration, we still followed the classic method and regarded dispositional optimism as unidimensional. Future studies are strongly suggested to discuss the influences of optimism and pessimism on subjective well being respectively. Thirdly, patients with Head and Face Burns or severe burns were not recruited in adherence to the recommendation of the ethics committee. Nevertheless, we suppose that an individual's mental structure resembles a spring, with resilience only working when the pull strength is not too powerful. An important issue to verify in future studies is whether dispositional optimism and psychological resilience can protect individuals who suffer from severe psychological trauma or fatal frustrations.
